# A Clinical Conundrum in Anorexia Nervosa – Starvation Hepatitis vs Refeeding Hepatitis

**DOI:** 10.1192/bjo.2025.10712

**Published:** 2025-06-20

**Authors:** Jwalamukhi Chidambaram Thirugnanam, Nikola Kern

**Affiliations:** South London and Maudsley NHS Foundation Trust, London, United Kingdom

## Abstract

**Aims:** Anorexia nervosa (AN) is a severe eating disorder, with a lifetime prevalence estimated to be 0.3–0.9%. Transaminase elevations are common in patients during hospital admission, reaching a prevalence of 43%. Although usually caused by refeeding, prolonged starvation can also cause an exacerbation of liver enzyme levels. It is important to differentiate between the two, as the treatment plans are quite different. We herein present a report of a 34-year-old lady with anorexia nervosa, who presented with extreme worsening of liver functions due to starvation hepatitis, while on admission for refeeding and her gradual recovery.

**Methods:** ‘A’ is a 34 year-old lady, who is known to specialist eating disorders team with long-standing history of anorexia nervosa, restrictive sub-type, in the background of coeliac disease. Body mass index (BMI) on admission was 11.4, and reported food intake till that point was less than 300 calories/day. Ward dietician started her on stage 2 refeeding menu – 750 calories, 25 g protein, 1350 ml fluid with appropriate thiamine, multivitamin and mineral cover. Liver function was mildly deranged (Alanine transaminase ALT 256 U/L, Gamma-glutamyl transferase activity GGT 38). ‘A’ struggled to eat on the ward, and over the next week deteriorated with LFT as follows – ALT 2362 U/L, AST 2288 U/L, GGT 88 U/L. Upon shifting to medical bed and failure of less restrictive options, ‘A’ was treated under the Mental Health Act with full nasogastric feeding with 1:1 supervision. Liver appeared normal on Ultrasound abdomen and serum electrolytes were mostly normal, ruling out refeeding hepatitis. Over the course of several weeks, as slowly BMI increased with improvement in nutrition, liver parameters improved with ALT dropping down to 346 U/L on day of transfer out of medical bed for psychological treatment.

**Results:** Starvation hepatitis, as in this case, appears when weight is at lowest with markedly elevated transaminases, normal liver appearance on radiological investigations. In this patient, BMI went to as low as 10 kg/m2 and expectedly, LFT derangements worsened, and improved on gaining weight.

**Conclusion:** Though anorexia nervosa has a plethora of medical complications, it is important to anticipate hepatitis as an important complication, and be aware of potential differential diagnoses including starvation hepatitis and refeeding hepatitis, which needs to be analysed carefully to delineate, and treat appropriately.

